# Adeno-associated viral serotypes produce differing titers and differentially transduce neurons within the rat basal and lateral amygdala

**DOI:** 10.1186/1471-2202-15-28

**Published:** 2014-02-18

**Authors:** Roopashri Holehonnur, Jonathan A Luong, Dushyant Chaturvedi, Anthony Ho, Srihari K Lella, Matthew P Hosek, Jonathan E Ploski

**Affiliations:** 1 School of Behavioral and Brain Sciences and the Department of Molecular & Cell Biology, University of Texas at Dallas, 800 West Campbell RD, Richardson, TX 75080, USA

## Abstract

**Background:**

In recent years, there has been an increased interest in using recombinant adeno-associated viruses (AAV) to make localized genetic manipulations within the rodent brain. Differing serotypes of AAV possess divergent capsid protein sequences and these variations greatly influence each serotype’s ability to transduce particular cell types and brain regions. We therefore aimed to determine the AAV serotype that is optimal for targeting neurons within the Basal and Lateral Amygdala (BLA) since the transduction efficiency of AAV has not been previously examined within the BLA. This region is desirable to genetically manipulate due to its role in emotion, learning & memory, and numerous psychiatric disorders. We accomplished this by screening 9 different AAV serotypes (AAV2/1, AAV2/2, AAV2/5, AAV2/7, AAV2/8, AAV2/9, AAV2/rh10, AAV2/DJ and AAV2/DJ8) designed to express red fluorescent protein (RFP) under the regulation of an alpha Ca2+/calmodulin-dependent protein kinase II promoter (αCaMKII).

**Results:**

We determined that these serotypes produce differing amounts of virus under standard laboratory production. Notably AAV2/2 consistently produced the lowest titers compared to the other serotypes examined. These nine serotypes were bilaterally infused into the rat BLA at the highest titers achieved for each serotype and at a normalized titer of 7.8E + 11 GC/ml. Twenty one days following viral infusion the degree of transduction was quantitated throughout the amygdala. These viruses exhibited differential transduction of neurons within the BLA. AAV2/7 exhibited a trend toward having the highest efficiency of transduction and AAV2/5 exhibited significantly lower transduction efficiency as compared to the serotypes examined. AAV2/5′s decreased ability to transduce BLA neurons correlates with its significantly different capsid protein sequences as compared to the other serotypes examined.

**Conclusions:**

For laboratories producing their own recombinant adeno-associated viruses, the use of AAV2/2 is likely less desirable since AAV2/2 produces significantly lower titers than many other serotypes of AAV. Numerous AAV serotypes appear to efficiently transduce BLA neurons, with the exception of AAV2/5. Taking into consideration the ability of certain serotypes to achieve high titers and transduce BLA neurons well, in our hands AAV2/DJ8 and AAV2/9 appear to be ideal serotypes to use when targeting neurons within the BLA.

## Background

Numerous genetic manipulation strategies have been developed to study complex interactions between gene expression and behavior. These strategies are essential to study the role and function of genes in complex systems such as the brain [1-5]. Gene manipulation strategies are designed to alter the genetic makeup in certain cells or tissue types either by over- expressing or knocking down specific genes. Some of the common methods currently being used for this purpose are traditional transgenic animal technology [6] and the relatively newer method utilizing recombinant viral vectors [7].

Within neuroscience, recombinant viral vector technology allows the targeting of localized populations of neurons or other cell types within specific parts of the nervous system. Recombinant viruses engineered to harbor transgenes of interest can be infused directly into desired brain regions where the virus can then transduce cells within the region of the infusion to deliver its transgene cargo thereby genetically modifying the targeted cells [8]. One of the greatest benefits of this technology is that it allows the genetic manipulation to be introduced at virtually any point in the organism’s life span quickly and easily. This is especially valuable in behavioral neuroscience research since the virus/transgene can be introduced before or after behavior experiments; thereby allowing it to be determined how the precise genetic manipulation specifically modifies specific aspects of the organism’s behavior [3,9-11].

The Adeno-Associated Virus (AAV) is an ideal virus to use for *in vivo* purposes since it is well tolerated *in vivo* and can be easily produced within the laboratory at titers necessary for this use. AAV does not cause disease and does not induce a significant inflammatory or immune response *in vivo*[12-20]. This class of viruses also exhibits stable and long lasting transgene expression and a wide range of infectivity [17,21]. Numerous AAV serotypes have been isolated from adenoviral stocks [22,23], from humans [24-26] and from primate tissues [24-26]; some AAV serotypes have also been engineered by directed evolution [27-29]. These AAV serotypes possess three different capsid proteins: Virion Proteins 1, 2, 3 (VP1, VP2 and VP3). Of these, VP1 is the largest capsid protein of the three, while VP2 and VP3 are produced through differences in splicing and translation initiation. Variations in the capsid protein amino acid composition among the AAV serotypes contribute to each serotype’s ability to transduce particular cell types [30-33].

Among the different AAV serotypes, AAV2 has been the most commonly used in neuroscience research, however; recent studies have determined that the transduction efficiency of this serotype is sub‒optimal for some brain cells and tissues [33-35]. These studies are incredibly important because they underscore that the choice of virus/serotype should be made wisely since all AAV serotypes may not necessarily transduce the target cell/tissue intended. Unlike traditional transgenic animal technology that typically genetically manipulates every cell or specific cell type within the whole organism, recombinant viral vector technology must be optimized to achieve transduction of as many cells as possible within the desired region. Optimal transduction is dependent on the viral titers that can be achieved and the efficiency of a particular virus for transducing a particular cell type.

Here we examined the ability of 9 different AAV serotypes (seven naturally occurring; AAV1, AAV2, AAV5, AAV7, AAV8, AAV9, AAVrh10 and two engineered though directed evolution; AAVDJ and AAVDJ8) in their ability to transduce neurons within the Basal and Lateral Amygdala (BLA). Targeting neurons within the BLA is of great interest for many in the neuroscience community due to the role of the BLA in emotional learning and memory [36-38], and psychiatric disorders [39,40]; importantly there are no previous reports on how effective these serotypes are at transducing neurons within the BLA.

## Results

### Pseudotyped viruses are essentially identical, except for their capsid proteins

**Figure 1:**
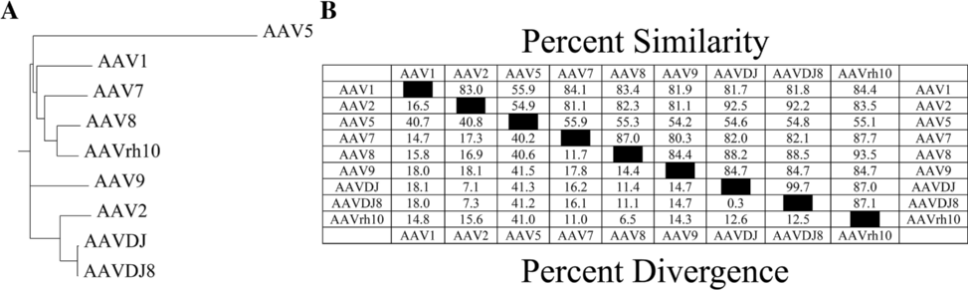
**Relative differences in amino acid composition among AAV serotype VP 1 proteins examined in this study. (A)**. Phenogram comparing graphically the relative differences among AAV serotype VP1 proteins. **(B)**. Percent similarity and divergence table comparing the relative differences among AAV serotype VP1 proteins.

To examine how AAV serotypes may differ in their intrinsic ability to transduce neurons within the Basal and Lateral Amygdala (BLA) an AAV2 genome plasmid harboring a red fluorescent protein gene, under the control of the neuron specific mouse αCaMKII promoter (AAV-RFP) [41,42], was pseudotyped into nine different AAV serotypes (AAV2/1, AAV2/2, AAV2/5, AAAV2/7, AAV2/8, AAV2/9, AAV2/rh10, AAV2/DJ and AAV2/DJ8). These pseudotyped viruses are essentially identical, except for their capsid proteins, VP1, VP2 and VP3. Sequence analysis of the largest capsid protein for each of these serotypes (i.e. VP1) highlights the degree to which these serotypes differ (Figure 1). Notably AAV5 differs the most relative to the other serotypes, exhibiting ~40% sequence divergence from these serotypes. The genetically engineered serotypes AAVDJ, and AAVDJ8 are very similar to each other; their closest relative is AAV2. Most of these serotypes differ by ~10-20%.

### Serotypes of AAV produce differing viral yields

**Figure 2:**
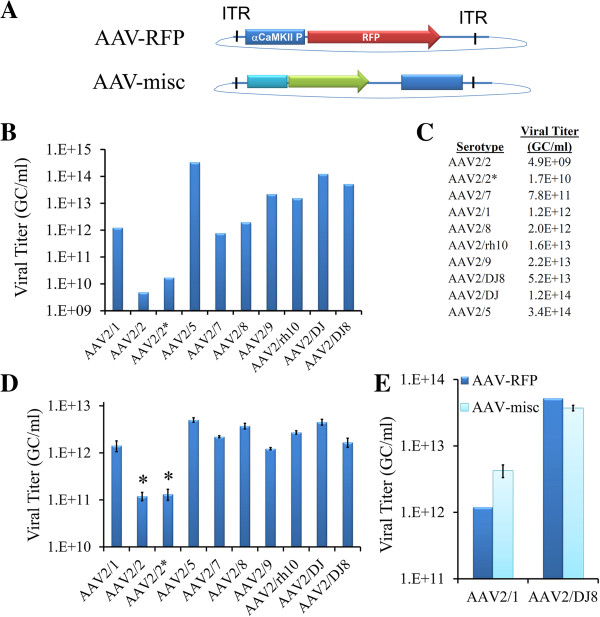
**AAV serotypes produce differing titers of virus following laboratory production. (A)**. Schematic diagram of AAV2 genome plasmids used in this study. AAV-RFP contains a red fluorescent protein gene (dsRed-Express) controlled by a mouse αCaMKII promoter that has been previously described [41]. AAV-misc has been previously described [43]. Both AAV2 genome plasmids contain genomes that span ~3300 bps (left inverted terminal repeat (ITR) to right ITR). **(B)**. Viral titers obtained following iodixanol purification. **(C)**. Viral titers obtained in **(B)**. displayed as a table. **(D)**. Viral titers obtained from crude viral lysates following small scale viral production. AAV2/2 produces significantly less packaged virus compared to the other serotypes examined. ANOVA *p* < .0001; Fisher’s Post-hoc test *p* < .05 for AAV2/2 and AAV2/2* compared to all other groups. *n* = 3. Error bars equal standard error of the mean (SEM). **(E)** AAV2/1 viral titers are ~ 10 fold less compared to AAV2/DJ8 viral titers following full scale production and iodixanol gradient purification. Viral titers for AAV-RFP viruses pseudotyped as AAV2/1 and AAV2/DJ8 (*n* = 1) are displayed next to viral titers for AAV-misc viruses pseudotyped at AAV2/1 (*n* = 2) and AAV2/DJ8 (*n* = 3). Error bars = SEM. Graphs displayed in **B**. **D**. and **E**. have logarithmic scales.

Following viral purification, the viruses were titered using a qRT-PCR based method to determine the number of viral genome copies per ml from DNase resistant viral particles. This method of titering provides the most accurate method to quantitatively compare viral yields from differing serotypes because it does not depend on viral transduction efficiency, which may naturally differ among serotypes. Following titering of these nine different viruses, it became apparent that there were large differences in the titers achieved for some of these serotypes (Figure 2B and C). AAV2/2 produced the lowest titer and AAV2/5 produced the highest titer. To ensure that the AAV2/2 titers were not due to an anomaly with the AAV2 serotype plasmid, AAV2/2 virus was produced again using a different AAV2 serotype plasmid obtained from another source (see methods) and it is referred to here as AAV2^*^. Both AAV2/2 and AAV2/2^*^ produced lower titers as compared to the other 8 serotypes produced. Because some variability in viral titer following large scale viral purification is to be expected, we next examined the ability of these serotypes to produce packaged virus using a crude viral lysate from a small scale experiment performed in triplicate (Figure 2D). This small scale viral production experiment yielded similar findings to the large scale experiment: AAV2/2 produced significantly lower viral yields compared to the other serotypes. AAV2/2 is not however the only virus that is prone to producing lower yields upon large scale viral production. For example, when the AAV2 genome plasmid harboring an RFP gene (AAV-RFP) was pseudotyped as AAV2/1, it produced titers that were on the lower end of viral yields as compared to the other serotypes that were purified (Figure 2B and C). When another AAV2 genome plasmid referred to here as AAV-misc was used to generate fully purified viruses that were pseudotyped as AAV2/1 and AAV2/DJ8, AAV2/DJ8 consistently produced viral titers in the range of 3E + 13 GC/ml, while AAV1 produced approximately 10 fold lower titers (Figure 2E).

### AAV serotypes differentially transduce neurons within the rat BLA

**Figure 3:**
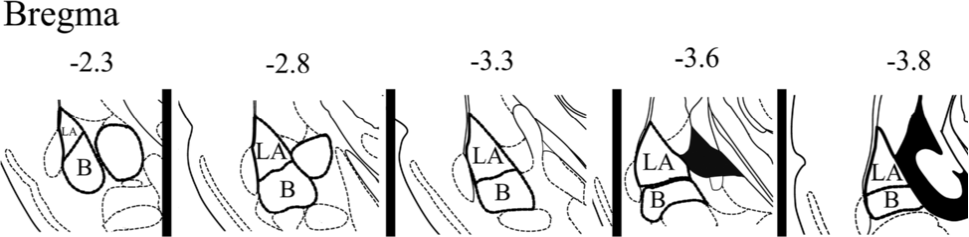
**Graphical depiction of the rat LA and B nuclei, (BLA) targeted within this study.** Anatomy across the anterior-posterior axis of the amygdala as previously described [44].

**Figure 4:**
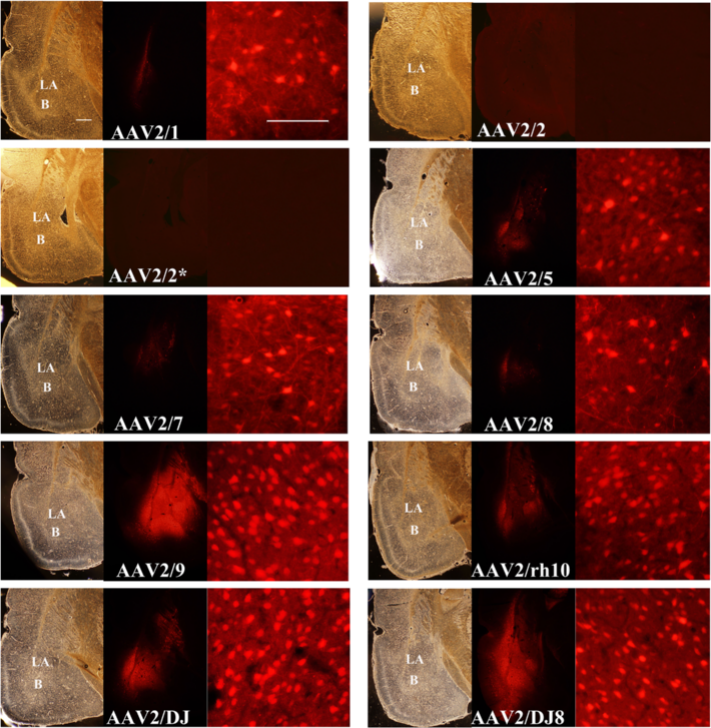
**Representative coronal images containing the amygdala (~ Bregma -3.3) depicting AAV-RFP viral transduction for selected AAV serotypes (RFP fluorescence) and associated bright field images.** In this experiment, AAV-RFP virus was pseudotyped as AAV2/1, 2/2, 2/5, 2/7, 2/8, 2/9, 2/rh10, 2/DJ, or 2/DJ8 and infused into the BLA at the highest titer achieved for each respective serotype (see Figure 2C). Images of coronal brain sections containing the amygdala are arranged for each serotype as follows: bright field image (scale bar = 500 μm), RFP image, and magnified view of a portion of the BLA region of the adjacent RFP image to allow visualization of the cellular detail (scale bar = 100 μm). LA = lateral nucleus of the amygdala, B = basal nucleus of the amygdala. For publication purposes, RFP images for AAV1, 5, 7, and 8 were slightly enhanced to increase clarity. AAV2/2 and AAV2/2* are the same serotype (see Methods) and neither of these viruses transduced any cells within the BLA at the respective titers achieved.

**Figure 5:**
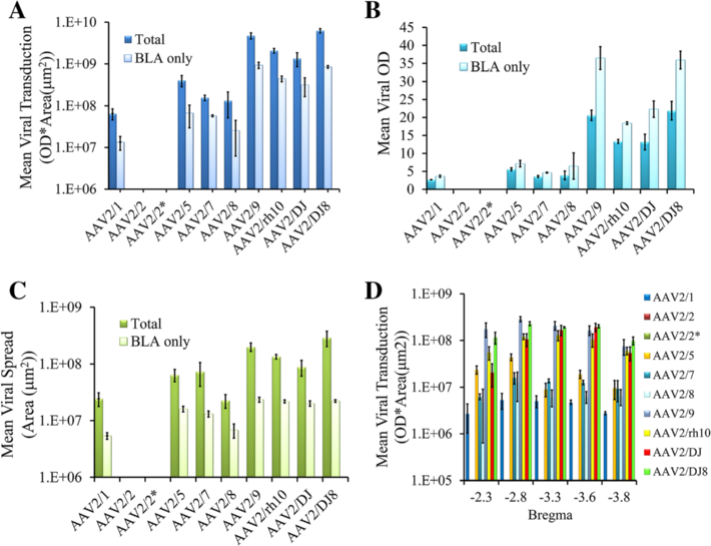
**Quantitation of viral transduction depicted in Figure**4**.** In this experiment, AAV-RFP virus was pseudotyped as AAV2/1, 2/2, 2/5, 2/7, 2/8, 2/9, 2/rh10, 2/DJ, or 2/DJ8 and infused into the BLA at the highest titer achieved for each respective serotype (see Figure 2C). **(A)**. Viral transduction (OD * area (μm^2^)) was quantified for each viral infusion and these data were averaged across infusions and reported as mean viral transduction. These measurements included measuring the entire amount of transduction (Total) and the viral transduction that was confined to the BLA (BLA only). These data are displayed side-by-side for each serotype. **(B)**. Viral OD was quantified for each viral infusion and these data were averaged across infusions and reported as mean viral OD. These measurements included measuring the OD for the entire amount of transduction (Total) and the OD for the viral transduction that was confined to the BLA (BLA only). These data are displayed side-by-side for each serotype. **(C)**. Viral spread (area (μm^2^)), was quantified for each viral infusion and these data were averaged across infusions and reported as mean viral spread. These measurements included measuring the viral spread for the entire viral transduction (Total) and the viral spread for the viral transduction that was confined to the BLA (BLA only). These data are displayed side-by-side for each serotype. **(D)**. For analysis of viral transduction across the anterior-posterior axis of the BLA, the amygdala was subdivided into 5 regions with respect to Bregma: -2.3 (-1.8 - -2.7); -2.8 (-2.7 - -3.1); -3.3 (-3.1 - -3.4); -3.6 (-3.4 - -3.7); 3.8 (-3.7 - -4.0) (see Figure 3) and the total BLA only transduction was quantified within each subregion. Graphs displayed in **A**. **C**. and **D**. have logarithmic scales. Error bars represent the standard error of the mean.

We next examined whether these 9 AAV serotypes differ in their ability to transduce neurons within the BLA (Figure 3). Because it was unknown what viral titer would prove optimal for infusion into the BLA, we first bilaterally infused each serotype at the highest titer achieved for each serotype (i.e. each serotype was infused at a different titer). Twenty one days following viral infusion, the rats were sacrificed, perfused and the amount of viral transduction was examined for each serotype throughout the entire amygdala (Bregma -1.80 to -4.16) (Figure 4). Since multiple viral particles are capable of transducing individual cells within the target region, and therefore able to deliver a differential number of copies of the RFP transgene to the target cells, it is necessary to measure the intensity of RFP fluorescence (optical density, OD) and the spread of viral transduction (area of cells containing RFP expression) to adequately measure efficiency of viral transduction among differing serotypes. Additionally, because targeted viral infusions within the rodent brain, even under the best circumstances will not entirely localize viral transduction to the intended target region, we specifically measured the intensity and spread of RFP fluorescence for the entire transduced region in each examined amygdala-containing coronal slice (see Methods) and we refer to these measurements as “Total”. We also measured the intensity and spread of RFP fluorescence within the BLA to determine “BLA only” measurements. The Total and BLA only measurements are graphed side-by-side (Figure 5).

In this experiment it became clear that high viral titers did not guarantee high viral transduction. For example, AAV2/5 had the highest titer of any serotype produced, but transduced BLA neurons modestly with respect to many of the other serotypes examined. At the titers achieved for AAV2/2 and AAV2/2^*^, no viral transduction was observed within the BLA. Serotypes AAV2/DJ8 and AAV2/9 possessed the highest level of transduction of BLA neurons in this experiment. Notably there was a high level of viral transduction that spanned the entire anterior-posterior axis of the BLA (Figure 5D), without any noticeable damage to the tissue.

**Figure 6:**
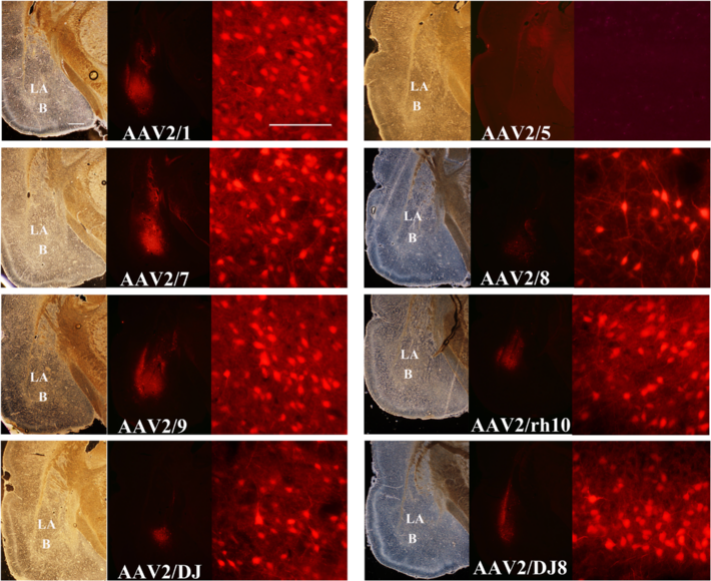
**Representative coronal images containing the amygdala (~ Bregma -3.3) depicting AAV-RFP viral transduction for selected AAV serotypes (RFP fluorescence) and associated bright field images.** In this experiment, AAV-RFP virus was pseudotyped as AAV2/1, 2/5, 2/7, 2/8, 2/9, 2/rh10, 2/DJ, or 2/DJ8 and infused into the BLA at the titer of 7.8E + 11 GC/ml. Images of coronal brain sections containing the amygdala are arranged for each serotype as follows: bright field image (scale bar = 500 μm), RFP image, and magnified view of a portion of the BLA region of the adjacent RFP image to allow visualization of the cellular detail (scale bar = 100 μm). LA = lateral nucleus of the amygdala, B = basal nucleus of the amygdala. No transduction was observed for AAV5 in this experiment.

**Figure 7:**
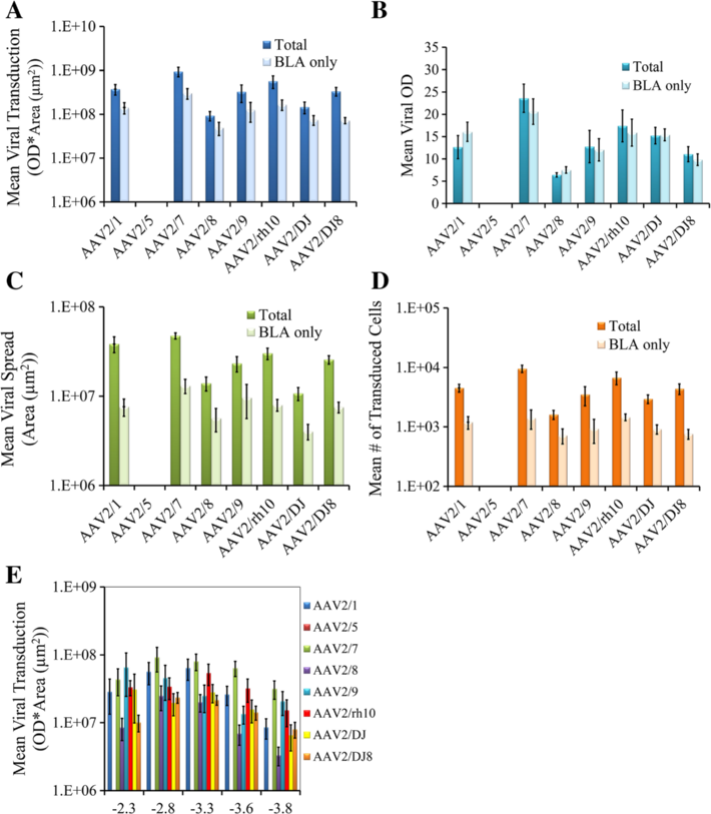
**Quantitation of viral transduction depicted in Figure**6**.** In this experiment, AAV-RFP virus was pseudotyped as AAV2/1, 2/5, 2/7, 2/8, 2/9, 2/rh10, 2/DJ, or 2/DJ8 and infused into the BLA at the titer of 7.8E + 11 GC/ml. **(A)**. Viral transduction (OD * area (μm^2^)) was quantified and depicted using methods described in Figure 5. **(B)**. Viral OD was quantified and depicted using methods described in Figure 5. **(C)**. Viral spread (area (μm^2^)), was quantified and depicted using methods described in Figure 5. **(D)**. The number of transduced cells was quantified for each viral infusion and these data were averaged across infusions and reported as mean number of transduced cells. These measurements included measuring the number of transduced cells for the entire viral transduction (Total) and the number of transduced cells for the viral transduction that were confined to the BLA (BLA only). **(E)**. For analysis of viral transduction across the anterior-posterior axis of the BLA, the amygdala was subdivided into 5 regions with respect to Bregma: -2.3 (-1.8 - -2.7); -2.8 (-2.7 - -3.1); -3.3 (-3.1 - -3.4); -3.6 (-3.4 - -3.7); 3.8 (-3.7 - -4.0) (see Figure 3) and the total BLA only transduction was quantified within each subregion. Graphs displayed in **A**. **C**. **D**. and **E**. have logarithmic scales. Error bars represent the standard error of the mean.

In our first experiment (above), we infused the viruses at differing titers, however; to examine AAV serotype transduction efficiency of BLA neurons quantitatively it was necessary to infuse the viruses at the same viral titer. In the next experiment, these serotypes were bilaterally infused into the rat BLA at a normalized titer of 7.8E + 11 GC/ml and 21 days following viral infusion the rats were sacrificed and the degree of transduction was quantitated throughout the amygdala as described above (Figures 6 and 7). In addition the number of transduced cells was determined for the entire viral transduction (total) and for transduction confined to the BLA (BLA only) (Figure 7). It was necessary to use a longer exposure time to capture the viral transduction images for this experiment since generally fewer viral particles were infused into the BLA as compared to the previous experiment. These data depicted in Figures 6 and 7, therefore, cannot be directly compared to data depicted in Figures 4 and 5 (see Methods).

These viruses exhibited differential transduction of neurons within the BLA. It is of interest that AAV2/5 (total and BLA only) exhibited the lowest viral transduction efficiency (mean viral transduction and number of transduced cells) as compared to the other 7 serotypes examined (Kruskal-Wallis test, Dunn-Bonferroni post hoc; p < .05). These findings were unambiguous since AAV2/5 consistently did not transduce cells within or around the BLA across replicates within this experiment. AAV serotypes 2/1, 2/7, 2/8, 2/9, 2/rh10, 2/DJ, and 2/DJ8 exhibited relatively comparable levels of transduction efficiency, with AAV2/7 exhibiting a trend for the highest level of transduction. Tables containing the p-values from statistical comparisons among the serotypes for mean viral transduction (total and BLA only), mean viral spread (total and BLA only) and mean number of transduced cells (total and BLA only) are provided in (Additional file 1: Table S1). AAV2/2 and AAV2/2* were not included in this experiment because the titers achieved for these viruses did not exhibit transduction of BLA neurons and these titers were below 7.8E + 11 GC/ml.

**Figure 8:**
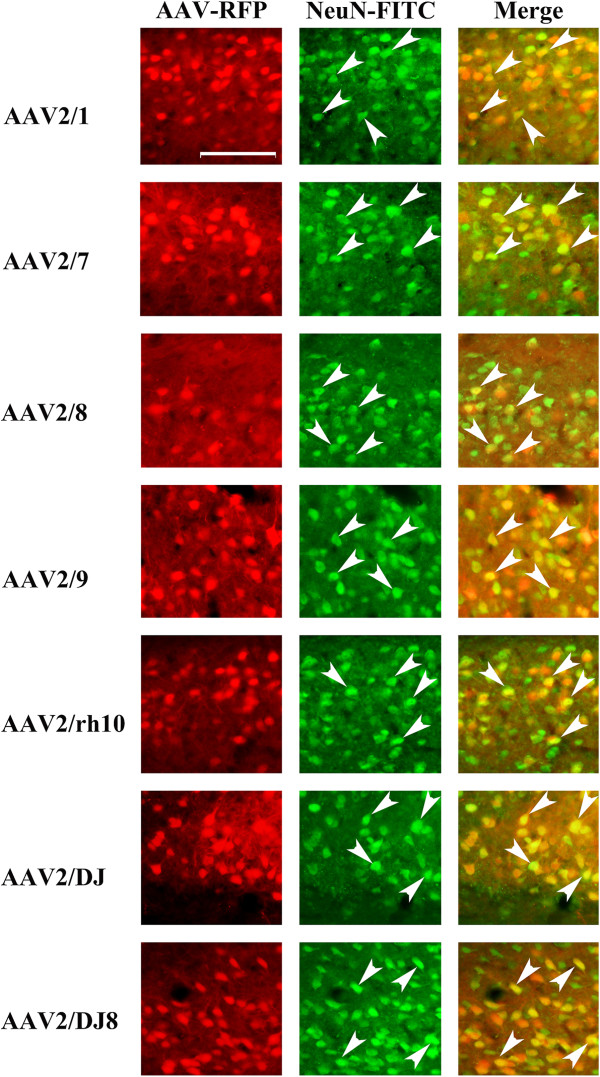
**Representative coronal images containing the BLA (~ Bregma -3.3) depicting AAV-RFP viral transduction for selected AAV serotypes (RFP fluorescence) and NeuN immunohistochemistry (FITC fluorescence).** In this experiment, AAV-RFP virus was pseudotyped as AAV2/1, 2/5, 2/7, 2/8, 2/9, 2/rh10, 2/DJ, or 2/DJ8 and infused into the BLA at the titer of 7.8E + 11 GC/ml. Images of coronal brain sections containing the BLA are arranged for each serotype as follows: AAV-RFP image (scale bar = 100 μm), NeuN-FITC image and an image of these two previous images merged. Arrows point to a subset of NeuN positive cells that are also AAV-RFP positive.

**Figure 9:**
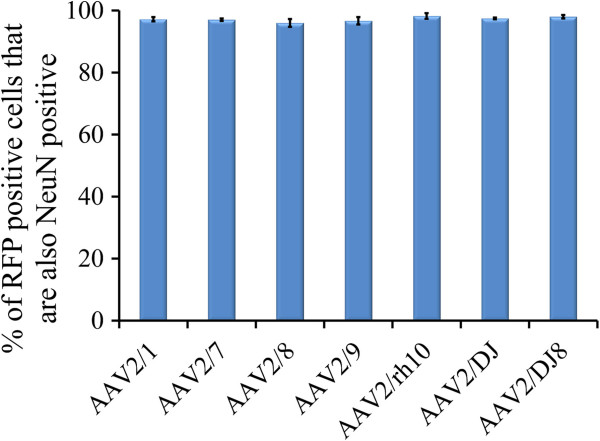
**Quantitation of data presented in Figure**8**to determine the percent of AAV-RFP positive cells that are also NeuN positive.** Error bars represent the standard error of the mean.

**Figure 10:**
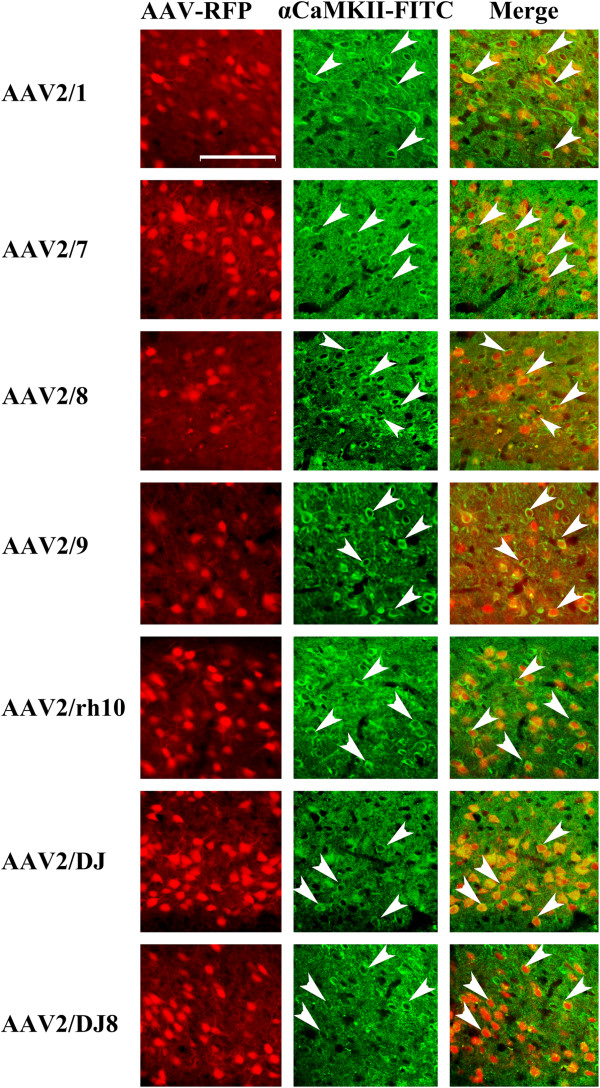
**Representative coronal images containing the BLA (~ Bregma -3.3) depicting AAV-RFP viral transduction for selected AAV serotypes (RFP fluorescence) and αCaMKII immunohistochemistry (FITC fluorescence).** In this experiment, AAV-RFP virus was pseudotyped as AAV2/1, 2/5, 2/7, 2/8, 2/9, 2/rh10, 2/DJ, or 2/DJ8 and infused into the BLA at the titer of 7.8E + 11 GC/ml. Images of coronal brain sections containing the BLA are arranged for each serotype as follows: AAV-RFP image (scale bar = 100 μm), αCaMKII-FITC image and an image of these two previous images merged. Arrows point to a subset of αCaMKII positive cells that are also AAV-RFP positive.

**Figure 11:**
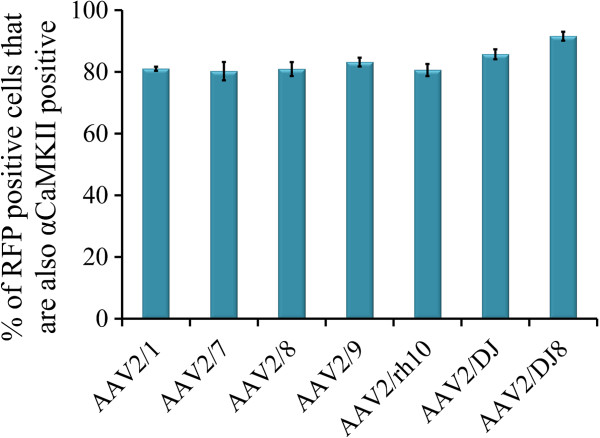
**Quantitation of data presented in Figure**10**to determine the percent of AAV-RFP positive cells that are also αCaMKII positive.** Error bars represent the standard error of the mean.

The viruses utilized within this study were designed to express RFP from a neuron specific mouse αCaMKII promoter and therefore regardless of the cell type these viruses transduce, only αCaMKII positive neurons would likely express the RFP. To examine if this prediction held true, we performed immunohistochemistry on BLA containing coronal slices that were transduced with serotypes 2/1, 2/7, 2/8, 2/9, 2/rh10, 2/DJ, and 2/DJ8 since these serotypes exhibited BLA transduction in the previous experiment. The proportion of RFP expressing cells that were also NeuN positive was examined and it was determined that ~98% of RFP expressing cells were also NeuN positive indicating that the RFP signal predominantly came from neurons (Figures 8 and 9). These data are consistent with the fact, that for the AAV serotypes examined thus far within the rodent central nervous system, all of them predominantly transduce neurons [34,35,45-49]. Next the proportion of RFP expressing cells that was also immuno positive for αCaMKII protein was examined. It was determined that ~85% of RFP expressing cells were also αCaMKII positive (Figures 10 and 11).

## Discussion

The impetus for this study was based upon the observation that for AAV-mediated gene delivery to the BLA to be truly effective, a large number of pertinent cells would need to be transduced and this would require using an AAV serotype that efficiently transduces the cell types of interest within this region, at titers that can be realistically obtained. Numerous studies have previously reported that the degree of viral spread and transduction within particular brain regions and cells can vary depending on AAV serotype [33,34,46,50]. For example, one study discovered that AAV2/8 displayed greater transduction and spread compared to AAV2/2 and AAV2/1 in numerous regions within the mouse brain [46]. Another study found that AAV2/2 did not transduce the dentate gyrus or CA1 effectively, which was in contrast to AAV2/1. Instead, AAV2/2 infected the hilar region of the dentate gyrus [34]. These studies underscore the importance of determining which AAV serotype most effectively transduces the cell population/brain region of interest since some serotypes may not transduce all cell types or brain regions well. However these previous studies only examined a few serotypes and none of these previous studies compared the abilities of differing AAV serotypes to transduce BLA neurons. Because of this it was necessary to screen AAV serotypes in their ability to transduce neurons of the BLA. We specifically sought to determine which serotypes are optimal for transducing αCaMKII positive neurons within the BLA since these neurons are critical for synaptic plasticity relevant for amygdala dependent learning and memory [36,38,51]. To accomplish this, we utilized an AAV2 genome plasmid that was designed to express RFP under the control of an αCaMKII promoter, to direct the expression of RFP predominantly in glutamatergic excitatory neurons [41,42]. Our approach was collectively unique among studies comparing differences among AAV serotypes in transduction ability of the rodent brain in multiple ways. First, we examined 9 different serotypes, the most of any other study. Second, we are the first to report transduction efficiency of AAV2/DJ and AAV2/DJ8 within the rodent brain, and third, we examined neuron transduction utilizing an expression system that was controlled by an αCaMKII promoter, whereas all previous studies comparing serotypes within the rodent brain utilized expression systems that were controlled by general promoters (i.e. cytomegalovirus (CMV) and Chicken β-actin (CBA)) that drive expression in all cell types or pan-neuronal promoters (i.e. Synapsin) that drive expression in all neuronal types.

Our study revealed that of the 9 serotypes we examined, AAV2/2 consistently produced the lowest yield of virus when produced under standard laboratory conditions and it was incapable of transducing neurons within the BLA at the titer achieved (1.7E + 10 GC/ml). In contrast AAV2/9 and AAV2/DJ8 produced much higher titers (2.2E + 13 GC/ml and 5.2E + 13 GC/ml respectively), and possessed the greatest overall transduction of BLA neurons at these titers among the serotypes tested. Given its cited use, it is of great interest that AAV2/5 consistently produced the highest titer of any serotype examined, but it possessed a modest ability to transduce neurons within the BLA therefore making it a less favorable choice for targeting neurons within the BLA. Interestingly AAV2/5 exhibited not only the lowest transduction efficiency of BLA neurons, but it is also the serotype with the most divergent capsid protein sequences as compared to the other serotypes examined. The AAV5 VP1 capsid sequence is ~40% different as compared to the other 8 AAV serotypes, suggesting that AAV5′s significant VP1 sequence divergence is associated with reduced BLA neuronal transduction. AAV serotypes 2/1, 2/7, 2/8, 2/9, 2/rh10, 2/DJ, and 2/DJ8 exhibited comparable levels of transduction efficiency, with AAV2/7 exhibiting a trend for the highest level of transduction.

Using a small scale crude viral preparation it was revealed that AAV serotypes differ in their intrinsic ability to produce packaged virus; specifically that AAV2/2 produced significantly less virus as compared to the other serotypes examined. It is important to note that the differences in viral yield among the other serotypes to each other were relatively minor in this experiment. An important advantage of using the small scale crude viral preparation is that the viral yield is not dependent on the purification method (i.e. cesium chloride ultracentrifugation, iodixonal gradient or affinity purification) and therefore differences in the viral yield can be attributed to the virus itself. Additionally, there were relatively large differences in yields of the fully purified AAV-RFP virus among some of the serotypes. Moreover, when AAV-misc was pseudotyped as AAV2/1 and AAV2/DJ8, the AAV2/1 titers obtained were consistently ~10fold lower as compared to AAV2/DJ8. These differences might not be due to variations in the production of each serotype, but may be due to differences in how efficiently differing serotypes are purified using the iodixonal gradient method.

## Conclusion

In conclusion this study determined that AAV serotypes differ in their ability to produce virus and differ in their ability to transduce neurons within the BLA. Based on the ability of AAV2/DJ8 to produce high titers and transduce neurons within the BLA exceptionally well at these titers, our lab has begun to routinely utilize AAV2/DJ8 in our ongoing experiments designed to target the BLA.

## Methods

### Subjects

Adult male Sprague Dawley rats (*Charles River Laboratories*) weighing 300-400 g were housed individually and maintained on a 12 hr light / dark cycle. Food and water were provided *ad libitum* throughout the experiment. Animal use procedures were in accordance with the National Institutes of Health Guide for the Care and Use of laboratory animals and were approved by the University of Texas at Dallas Animal Care and Use Committee.

### Large scale AAV production/purification

An AAV2 genome plasmid harboring an RFP gene controlled by a mouse αCaMKII promoter (AAV-RFP) was packaged into functional viruses that were pseudotyped with different AAV capsid proteins to produce AAV serotypes 2/1, 2/2, 2/5, 2/7, 2/8, 2/9, 2/rh10, 2/DJ and 2/DJ8. Pseudotyped viruses were produced using a triple-transfection, helper-free method, and the resultant viruses were purified on an iodixanol step gradient, as previously described [43,52]. For producing each virus, AAV-293 cells (Agilent Technologies) were seeded in 5×15 cm plates 24 hours before transfection so that at the time of transfection the cells would be 80-85% confluent. At the time of transfection 135 μg of pRC (serotype plasmid), 135 μg of pHelper and 135 μg of AAV2 genome plasmid harboring a red fluorescent protein gene (dsRed-Express) controlled by a mouse αCaMKII promoter (Addgene plasmid 22908) [41] were used per each calcium phosphate mediated transfection. The culture media was replaced 6 hours post-transfection with pre-warmed standard growth media. At Seventy-two hours post-transfection the cells were harvested by centrifugation (1500 g for 15 min), the supernatant was discarded and the cells were re-suspended in 8 ml of freezing buffer (0.15 M NaCl, 50 mM Tris, pH 8.0) and stored at -80°C until purification. For virus purification, cells were lysed by two freeze/thaw cycles in dry ice-ethanol and 42°C water baths. Five hundred units of benzonase (Novagen) was added to the lysate and incubated for 30 minutes at 37°C. The lysate was clarified by centrifugation at 3700 g for 20 min and the supernatant was applied to an iodixanol gradient column (15%, 25%, 40% and 57% iodixanol). The columns were centrifuged at 50,000 rpm for 3 hr 20 min at 10°C in a Type 70 Ti rotor (Beckman Coulter). After centrifugation the 40% iodixanol layer was extracted and transferred to a conical tube and diluted with 25 ml of PBS-MK (1×PBS without calcium or magnesium, 1 mM MgCl_2_, 2.5 mM KCl) and concentrated using Amicon Ultra-15 Centrifugal Filter Units (Millipore) to ~ 1 ml. This viral concentrate was diluted again with 10 ml of PBS-MK and concentrated to a final volume of 100 μl and stored at 4°C until use. All plasmids used were purified using Qiagen Endofree plasmid purification kits following the manufacturer’s instructions. AAV packaging plasmids (pRC) for different serotypes were obtained from: AAV1, AAV5 (Nicholas Muzyczka, University of Florida), AAV-2 (Agilent Technologies), AAV2*, AAVDJ and AAVDJ8 (Cell BioLabs, Inc), AAV7, AAV8, AAV9, AAVrh10 (Penn Vector Core, University of Pennsylvania). AAV2 and AAV2* are the same serotype but the AAV2 packaging plasmid was obtained from a different source (above). AAV Helper plasmid (pHelper) was obtained from (Agilent Technologies). Since calcium phosphate transfections are sensitive to the pH of the 2×HBS solution, we prepared 2×HBS at different pH (pH 7.04, 7.06, 7.08, 7.10 and 7.12) and tested them in small scale transfections in a 6 well plate with a GFP reporter plasmid to determine the optimal 2×HBS solution to use for transfection. Transfections to produce AAV serotypes 1 and 5 were performed as described above with the exception that 270 μg of the AAV1 and AAV5 serotypes plasmids were transfected and the pHelper plasmid was not transfected since the packaging plasmid for these serotypes contained both capsid sequences and helper sequences required for making AAV viruses. AAV-misc is essentially the same AAV shRNA genome plasmid that has been previously described [43] with the exception that the U6 based shRNA expression cassette has been replaced with a H1 based shRNA expression cassette. AAV-misc based viruses were produced and purified as described above.

### Viral titering

Purified AAV viruses were titered using a quantitative-PCR based titering method. Five μl of purified viruses were treated with 20 Units of DNase I (Roche) at 37°C for 30 min and then further diluted in (10 mM Tris pH 7.4, 10 μg/ml of yeast tRNA solution (Ambion)) to make the final concentration of virus a 1:10,000 dilution with respect to the undiluted virus. Five μl of the diluted virus was used per a standard 20 μL Taqman PCR assay (Applied Biosystems). One μl of a 20X Taqman custom RFP Primer/Probe for AAV-RFP (RFP FP: AGCGCGTGATGAACTTCGA,

RFP RP: GCCGATGAACTTCACCTTGTAGAT, RFP Probe: 6FAM-ACCCAGGACTCCTCC) (as previously described [53]) or GFP Primer/Probe (ID# Mr04329676_mr, Invitrogen) for AAV-misc was used per reaction. Samples were prepared and loaded onto a 96 well plate in triplicate and quantitated using a CFX96 Real-time PCR system (BioRad) using the standard cycling parameters specified by Applied Biosystems. AAV-RFP and AAV-misc viral genome copies were quantitated based on a standard curve prepared by serially diluting the pAAV-αCaMKII-RFP and pAAV-misc plasmids respectively in (10 mM Tris pH 7.4, 10 μg/ml of yeast tRNA solution) across eight, three fold serial dilutions ranging from 1 × 10^3^ to 3 × 10^6^ copies of the viral genome plasmid per PCR reaction (as described above). Final viral titers were computed based on the standard curve and reported as genome copies GC/ml.

### Viral titering of small scale viral crude lysate

Small scale transfections for AAV-RFP serotypes were carried out in 12 well plates using standard Lipofectamine transfection protocol (Life Technologies). Twenty four hr before transfection, AAV-293 cells were seeded in 12 well plates in AAV-293 growth media without antibiotics so that on the day of transfection the cells were 90-95% confluent. At the time of transfection, .53 μg of pRC (serotype plasmid), .53 μg of pHelper and .53 μg AAV2 genome plasmid were transfected into each well following the manufacturer’s instructions (Invitrogen). Seventy two hours post transfection the cells were harvested by centrifugation at 1500 g for 15 min and the cell pellet was re-suspended in 36.5 μl of freezing buffer. The cells were then lysed by two freeze/thaw cycles in a dry ice-ethanol bath and a 42°C water bath. Two and one half units of benzonase (Novagen) were added to the lysate and incubated for 30 min at 37°C. The lysate was then clarified by centrifugation at 3700 g for 20 min and the supernatant was used for PCR based viral titering. The partially purified viruses were treated with 20 Units of DNase I (Roche) at 37°C for 30 min and then diluted 1:1000 in (10 mM Tris pH 7.4, 10 μg/ml of yeast tRNA solution) and were titered similarly as the large scale AAV virus titering. The titers were reported as GC/ml. For this experiment all samples were processed together in triplicate to eliminate inter-experiment variability.

### Basolateral complex (BLA) viral infusions

Thirty-three gauge custom made infusion cannula (*C315G,* Plastics One) were inserted into ~20 inch long polyethylene tubing (I.D. 0.015 in, O.D. 0.043 in, wall thickness 0.0140 in) (A-M systems, Inc*.*). These tubes were backfilled with sesame oil and then attached to 2 μl, 23-gauge (88500) stainless steel Hamilton syringes (Hamilton Company). Under a mixture of Ketamine (100 mg/kg) and Xylazine (10.0 mg/kg) anesthesia, rats were stereotaxically implanted bilaterally with a 33 gauge infusion cannula (described above) targeting the BLA [AP -2.9, ML ±5.2, DV -8.6]. For experiments depicted in Figures 4 and 5, 1.4 μl of the highest titer for each serotype (see Figure 2C), were bilaterally infused for 15 minutes at a rate of 0.09 μl/min. For experiments depicted in Figures 6 and 7, the viral titers for each serotype were adjusted to 7.8E + 11 GC/ml using PBS-MK and 1 μl was bilaterally infused for 15 min at the rate of 0.07 μl/min. Following infusions, the infusers were left in for 10 additional min to allow for diffusion of the virus away from the cannula after which they were withdrawn and the incision was closed using 9 mm wound clips (Mikron Precision, Inc.). The rats were allowed one week for recovery and the wound clips were removed using a wound clip remover (Mikron Precision, Inc.). Three weeks post infusion, the rats were anesthetized with an overdose of chloral hydrate (250 mg/kg) and then perfused with phosphate-buffered saline (1× Phosphate buffer, 150 mM NaCl) and 10% buffered Formalin (Fisher Scientific). The brains were fixed in 10% formalin for 4-5 hours followed by cryoprotection in 1×PBS pH7.4, 30% sucrose for 4-6 days.

### Imaging and quantification of viral transduction

Following cryoprotection, the brains were frozen and 40 μm coronal sections were obtained using a cryostat which included the entire amygdala (Bregma -4.16 to -1.80). Every alternate section was placed on superfrost slides (Fisherbrand) typically yielding ~40 sections per amygdala. The remaining slices were stored in a solution of PBS-MK with 0.1 mM sodium azide. The sections on superfrost slides were imaged using an Olympus IX51 inverted fluorescent microscope under RFP and bright field filters and the images were acquired using an Olympus DP72 Digital Camera and Cellsens software (Olympus). Approximately 2500 images were taken and analyzed for this project. For experiments depicted in Figures 4 and 5, all fluorescence images were taken under the same exposure conditions of 67 ms at 20X magnification. For experiments depicted in Figures 6 and 7, all fluorescence images were taken under the same exposure conditions of 1 s at 20X magnification. As a result, images between these experiments cannot be compared directly. All ~40 sections per amygdala were quantified for viral transduction efficiency using Image-J software to specifically measure spread of transduction (Area (μm^2^)), mean red fluorescence intensity per transduced region (optical density (OD)), and mean viral transduction (Area*OD). For experiments depicted in Figures 6 and 7, in addition to these three measurements, the mean number of cells transduced was also determined. Because targeted viral infusions within the rodent brain even under the best circumstances will not entirely localize viral transduction to the intended target region, all 4 of these measures were taken for the total viral transduction within each imaged coronal brain slice (designated as Total within the figures), and the viral transduction which occurred only within the borders of the BLA (designated as BLA only within the figures). The total viral transduction (Area*OD) per slice was calculated by subtracting the background mean optical density (OD) from the mean OD of the total area transduced in each slice to create the OD of transduction per slice. This value was multiplied by the total area transduced within each slice, measured in μm^2^ to yield a measure of total viral transduction per slice. The total viral transduction per amygdala was recorded as the sum of all measures of total viral transduction per slice per amygdala and the total viral transduction per serotype was reported as the mean total viral transduction of all amygdala infusions per serotype demonstrating transduction. OD of total transduction was calculated for every slice and averaged across all slices per amygdala to yield total OD of transduction per amygdala and this value was averaged among all infusions of a particular serotype demonstrating transduction to yield mean total OD of viral transduction per serotype. The total spread of transduction per amygdala was recorded as the sum of total area transduced in all slices per transduced amygdala and this value was averaged among all infusions of a particular serotype demonstrating transduction to yield mean total viral spread per serotype. For BLA only viral transduction measurements, the OD and viral spread were calculated as above with the exception that the measurements were limited to the boundaries of the BLA in each slice and these measurements (BLA only) are reported side by side with the Total measurements within the figures. In experiments depicted in Figures 4 and 5, approximately 40 slices per amygdala were analyzed and a total of 4 amygdala per serotype were analyzed, with the exception of AAV-DJ which 5 amygdala were analyzed. A total of 2-3 rats per serotype were analyzed. For experiments depicted in Figures 6 and 7, in addition to the above mentioned measurements we also counted the number of transduced cells. To quantify the number of cells transduced, images were imported into ImageJ and converted to grayscale 8 bit images and the background subtraction function was applied. Next the threshold was set to 27 for the lower level and 255(max) for the higher level. The watershed function was applied and the cells within each image were counted for the entire image and cells only within the borders of the BLA were also counted (BLA only). The cell counts were summed across all sections that exhibited viral transduction per amygdala and cell counts per amygdala per serotype were averaged to give mean cell counts per serotype. For experiments depicted in Figures 6 and 7, approximately 40 slices per amygdala were analyzed and a total of 7-10 amygdala per serotype across 4-8 rats per serotype were analyzed (AAV2/1, AAV2/7, n = 7; AAV2/5, AAV2/8, AAV2/9, AAV2/rh10, n = 8; AAV2/DJ, n = 9; AAV2/DJ8, n = 10; n refers to number of amygdala per serotype analyzed). Non-parametric statistics were applied to evaluate statistical differences in transduction efficiency among serotypes examined using the Kruskal-Wallis test with a Dunn-Bonferroni post hoc test. Differences were considered significant if, p < 0.05 (uncorrected for multiple comparisons); however in many cases, significance was reached when correcting for multiple comparisons. Tables containing the p-values from statistical comparisons among the serotypes for mean viral transduction (total and BLA only), mean viral spread (total and BLA only) and mean number of transduced cells (total and BLA only) are provided in (Additional file 1: Table S1).

### Sequence analysis

To generate the phylogram comparing the VP1 capsid proteins for the AAV serotypes used in this study, the VP1 amino acid sequences for all of the serotypes were first aligned using ClustalOmega (EMBL-EBI) [54] using the default parameters. The resulting phylogenetic tree output file was then imported into Phylodendron software (version 0.8d, beta January 1999; http://iubio.bio.indiana.edu/treeapp/) to generate the phenogram. To produce the sequence similarity/divergence table for the AAV serotype VP1 capsid proteins, the VP1 amino acid sequences for all serotypes were imported into MegAlign Software version 3.05a (DNASTAR, Inc.) and aligned using the Clustal method and the pair-wise sequence distances were computed using the Clustal method with a PAM250 residue weight table.

### Immunohistochemistry

For immunohistochemistry (IHC), 1 μl of virus was bilaterally infused into the BLA at a titer of 7.8E + 11 GC/ml as described above. Twenty one days post infusion, the rats were anesthetized with an overdose of chloral hydrate (250 mg/kg) and then perfused with 4% Paraformaldehyde in 1 × PBS (pH = 7.4). Following brain extraction, the brains were fixed in 4% Paraformaldehyde in 1×PBS (pH = 7.4) for 4-5 hr, followed by cryoprotection in 1×PBS pH7.4, 30% sucrose for 4-6 days. Following cryoprotection, the brains were frozen and 40 μm coronal sections were obtained using a cryostat as described above. For NeuN IHC, brain slices were rinsed in 1×PBS for 10 min followed by incubation for 30 min with blocking buffer (1× PBS, 3% normal donkey serum, 0.3% Triton X-100). Next the slices were incubated overnight at 4°C with NeuN Antibody (1:500; MAB377 Millipore, Billerica, MA), diluted in blocking buffer. For the αCaMKII IHC, brain slices were rinsed in 1×PBS for 10 min followed by incubation for 60 min with blocking buffer (1X PBS, 5% normal donkey serum, 0.3% Triton X-100). Next the slices were incubated overnight at 4°C with αCaMKII Antibody (1:300; 05-532, Millipore), diluted in antibody dilution buffer (1XPBS, 1% BSA, 0.3% Triton X-100). For secondary antibody staining 1:500 dilution of fluorescein isothiocyanate (FITC) conjugated anti-Mouse IgG (AP192F; Millipore) was used and the slices were incubated for 2 hr at room temperature. The slides were coverslipped with Vectashield HardSet Mounting Medium (Vector Laboratories). IHC images were captured at 100× magnification using an Olympus BX51 upright fluorescence microscope with an Olympus DP71 Digital Camera and DP manager software. The number of AAV transduced (RFP) cells within the BLA that were also NeuN positive or αCaMKII positive was determined by quantifying the number of RFP cells that also co-localized with the NeuN or αCaMKII FITC signal within an area of 135173.56 μm^2^ from within the BLA of each slice examined. The area within each slice quantified typically contained ~ 65 RFP positive cells and a total of three slices were counted per serotype. Cells were manually quantified using Adobe Photoshop CS5 software and the colocation results were reported as a percentage of total RFP cells counted. Variability in co-localization results between slices was reported as standard error of the mean.

## Competing interests

The authors declare that they have no competing interests.

## Authors’ contributions

RH, JAL and SKL produced and titered the AAV used within this study. RH, JAL performed the stereotaxic viral infusions. RH, JAL, DC, AH, MPH prepared the coronal sections and imaged and quantified the viral transduction. RH performed the IHC and associated quantitation. RH and JEP conceived the study and participated in its design and coordination and drafted the manuscript. JEP performed the sequence analysis. All authors read, edited and approved the final manuscript.

## Supplementary Material

Additional file 1: Table S1 Tables containing the p-values from statistical comparisons among the serotypes for mean viral transduction (total and BLA only), mean viral spread (total and BLA only) and mean number of transduced cells (total and BLA only) for data depicted in Figures 6 and 7. Click here for file
